# Oligofructose alone and in combination with 2′fucosyllactose induces physiologically relevant changes in γ-aminobutyric acid and organic acid production compared to sole 2′fucosyllactose supplementation: an *in vitro* study

**DOI:** 10.1093/femsec/fiad100

**Published:** 2023-08-31

**Authors:** Peter Philip James Jackson, Anisha Wijeyesekera, Robert Adrian Rastall

**Affiliations:** Department of Food and Nutritional Sciences, University of Reading, Harry Nursten Building, Pepper Lane, Whiteknights, Reading RG6 6DZ, United Kingdom; Department of Food and Nutritional Sciences, University of Reading, Harry Nursten Building, Pepper Lane, Whiteknights, Reading RG6 6DZ, United Kingdom; Department of Food and Nutritional Sciences, University of Reading, Harry Nursten Building, Pepper Lane, Whiteknights, Reading RG6 6DZ, United Kingdom

**Keywords:** 2′fucosyllactose, bifidobacteria: prebiotic, GABA, gut–brain axis, oligofructose

## Abstract

We explored the potential for the prebiotic oligofructose and prebiotic candidate 2′fucosyllactose, alone and in combination (50:50 blend) to induce physiologically relevant increases in neurotransmitter (γ-aminobutyric acid, serotonin, tryptophan, and dopamine) and organic acid (acetate, propionate, butyrate, lactate, and succinate) production as well as microbiome changes using anaerobic pH-controlled *in vitro* batch culture fermentations over 48 h. Changes in organic acid and neurotransmitter production were assessed by gas chromatography and liquid chromatography and, bacterial enumeration using fluorescence *in situ* hybridization, respectively. Both oligofructose and oligofructose/2′fucosyllactose combination fermentations induced physiologically relevant concentrations of γ-aminobutyric acid, acetate, propionate, butyrate, and succinate at completion (all *P* ≤ .05). A high degree of heterogeneity was seen amongst donors in both neurotransmitter and organic acid production in sole 2′FL fermentations suggesting a large responder/nonresponder status exists. Large increases in *Bifidobacterium, Lactobacillus*, and *Bacteroides* numbers were detected in oligofructose fermentation, smallest increases being detected in 2′fucosyllactose fermentation. Bacterial numbers in the combined oligofructose/2′fucosyllactose fermentation were closer to that of sole oligofructose. Our results indicate that oligofructose and oligofructose/2′fucosyllactose in combination have the potential to induce physiologically relevant increases in γ-aminobutyric and organic acid production along with offsetting the heterogenicity seen in response to sole 2′fucosyllactose supplementation.

## Teaser

Exploring the potential of the prebiotic oligofructose and prebiotic candidate 2′fucosyllactose alone and in combination to induce physiologically relevant increases in neurotransmitter and organic acid production, and changes in bacterial population.

## Introduction

In recent years, there has been significant growth in interest in the bidirectional relationship existing between the gut and the brain—a term coined the gut–brain axis (Appleton 2018). There is an increasing body of evidence suggesting that the composition of the gut microbiota plays a key role in influencing mood state including emotional regulation, cognitive performance, and mental health, particularly anxiety and depression (Evrensel and Ceylan 2015). Within the gut, it is documented that several genera, species and strains of bacteria can produce a number of different metabolites associated with cognitive and mental state including neurotransmitters such as γ-aminobutyric acid (GABA), serotonin, and dopamine as well as organic acids—acetate, propionate, butyrate, lactate, and succinate (Cryan et al. 2020, Silva et al. 2020).

Prevalent GABA producers in the gut include several species and strains of *Bifidobacterium* and *Lactobacillus* and to a lesser extent *Bacteroides* (Strandwitz 2018, Strandwitz et al. 2019). Studies have reported that GABA can be produced via several pathways: first, by the conversion of arginine to ornithine to putrescine and GABA, resulting in the generation of succinate and finally propionate (Otaru et al. 2021). Second, it can be produced by the decarboxylation of glutamate (Barrett et al. 2012). GABA serves as an acid-resistance mechanism to survive the low pH of the intestinal environment (Strandwitz et al. 2019). However, it remains unclear whether GABA is able to cross the blood–brain barrier (Knudsen et al. 1988, Shyamaladevi et al. 2002), although it can be synthesized from acetate in the hypothalamus (Frost et al. 2014). Incidentally, there is accumulating evidence demonstrating that bacterial derived GABA more likely functions locally, being involved in neuronal excitability within the enteric nervous system along with contributing towards GI motility in the ilium and peristaltic reflux within the colon (Seifi et al. 2014, Seifi and Swinny 2019).

Another neurotransmitter, serotonin, is produced via the metabolism of tryptophan in the presence of enterochromaffin cells by various microorganisms including *Lactococcus, Enterococcus*, and Streptococcus (Kaur et al. 2019). Yet, it is estimated that ~90%–95% of tryptophan enters the kynurenine pathway resulting in the generation of either kynurenic or quinolinic acid (Gao et al. 2020, Muneer 2020). Of these two metabolites, kynurenic acid has been associated with neuroprotective properties and regulation of immune function, whereas increasing concentrations of quinolinic acid in the brain is associated with psychiatric and neurodegenerative disorders (Lugo-Huitron et al. 2013).

Organic acids such as acetate and lactate are predominately produced by *Bifidobacterium* via saccharolytic fermentation are reported to act as endocrine signalling molecules (Silva et al. 2020). Butyrate produced by bacteria including *Bacteroides* and *Roseburia*, either directly or as a result of cross-feeding on acetate and lactate, is speculated to aid in the expression of GABA receptors (Nankova et al. 2014). Furthermore, as documented by both (Reigstad et al. 2015, Dalile et al. 2019) organic acids likely aid in regulating the expression of tryptophan-5-hydroxylase 1 and tyrosine hydroxylase, enzymes involved in the rate-limiting step in the biosynthesis of several neurotransmitters by enteroendocrine cells.

The majority of glutamate and tryptophan, like many other amino acids, are absorbed and metabolized in the small intestine. Yet, a report from (Yao et al. 2016) estimated that on average between 7% and 10%, or 6–18 g/day, of dietary protein reaches the large intestine intact. This roughly equates to 3.4–6.3 mmol/kg total free amino acids entering the proximal and distal region of the colon. Currently, glutamate is estimated to be one of most abundant amino acids present in food, making up between 8% and 10% of all dietary protein consumed (van der Wielen et al. 2017). Given the sharp rise in high quality protein diets and glutamate rich foods (Beyreuther et al. 2007, Tennant 2018) it is estimated that daily consumption of glutamate ranges from 5 to 15 (12 average) g/day. In Asian countries this is largely due to increased consumption of free monosodium glutamate. Based on the assumption that 7%–10% of total dietary protein reaches the colon intact, and an average glutamate intake of 12 g/day, it can be speculated that somewhere in the region of 0.7–1.2 g of total dietary glutamate reaches the colon. Daily tryptophan consumption is estimated at ~900–1000 (950 average) mg/day (Richard et al. 2009) roughly equating to between 66.5 and 95 mg dietary tryptophan entering the colon.

The majority of research investigating changes in neurotransmitter production via manipulation of the microbiota primarily revolves around several strains of probiotics. This makes sense given the remarkable variability in neurotransmitter production seen in microorganisms, even those within the same genera and species (Kaur et al. 2019, Strandwitz et al. 2019). However, other means of targeted manipulation of the gut microbiota exist, including prebiotics and potential prebiotic oligosaccharide candidates. The most substantiated of all prebiotics are oligofructose (OF) and inulin, which belong to a class of nondigestible carbohydrates referred to as inulin-type fructans (ITF) (Mensink et al. 2015). To date, the ability of ITF to stimulate changes in gut microbiota composition has been substantially demonstrated, both *in vivo* and *in vitro*, across a wide array of dosages (Wang and Gibson 1993, Kolida et al. 2007, Vandeputte et al. 2017). Other oligosaccharides under consideration as prebiotics include human milk oligosaccharides (HMOs), a group of structurally diverse and complex unconjugated glycans present in human breast milk (Ninonuevo et al. 2006). The most common of these is 2′fuscosyllactose (2′FL), the first HMO to be produced on an industrial scale (Sprenger et al. 2017) and currently under investigation as a novel food ingredient and as a means of treating IBS and cognitive mental disorders (Al-Khafaji et al. 2020, Sans Morales et al. 2022). In comparison to ITF, the data on the ability of 2′FL to stimulate changes in microbial composition are mixed due to a limited number of clinical studies (Elison et al. 2016, Suligoj et al. 2020, Iribarren et al. 2021, Ryan et al. 2021).

Much remains unknown on whether supplementation with prebiotics/prebiotic candidates is enough to stimulate physiologically relevant increases in neurotransmitter production. This is in part due to the large heterogeneity existing between individual gut microbiotas and the need for an individual’s microbiota to possesses the required microorganisms. As a result, in this study we investigated whether it is possible for the prebiotic OF and prebiotic candidate 2′FL, singular and in combination, to stimulate physiologically relevant increases in neurotransmitter and organic acid production using pH-controlled *in vitro* batch culture fermentation over 48 h.

## Material and methods

### Materials

#### Prebiotic

The ITF used was OF (Orafti® P95, DP 3–9, average DP 4; BENEO-Orafti, Tienen, Belgium).

#### Prebiotic candidate

2′fucosyllactose is an HMO produced commercially via genetically modified yeasts and bacteria. 2′Fucosyllatose (96%–98% pure) is a fucosylated HMO composed of l-fucose, d-galactose, and d-glucose and was supplied by BENEO-Orafti.

#### Reagents

Unless otherwise stated all reagents used in this experiment were sourced from Merck, Gillingham, UK.

### *In vitro* batch culture fermentation

#### Faecal sample preparation

Ethical approval of collecting faecal samples from healthy volunteers was obtained from University of Reading Research Ethics Committee. Freshly voided faecal samples were obtained from five healthy adults aged between 18 and 40, who had not taken antibiotics for at least 4 months prior to the experiment, had no history of gastrointestinal disorders, were not regular consumers of prebiotics or probiotics and did not follow any restrictive diet. Faecal samples were diluted 1 in 10 (w/v) using 0.1 mol/l, pH 7.4 anaerobically prepared phosphate buffered saline (PBS, Oxoid, Hampshire, UK). Faecal samples were then homogenized in a stomacher (Seward, stomacher 80, Worthing, UK) for 120 s at 260 paddle-beats per min. Faecal slurry (5 ml) was immediately used to inoculate each batch culture vessel.

#### Glutamate and tryptophan-enriched basal batch culture nutrient medium

To make 1 l of basal nutrient medium, 2 g peptone water, 2 g yeast extract, 0.1 g NaCl, 0.04 g K_2_HPO_4_, 0.04 g KH_2_PO_4_, 0.01 g MgSO_4_.7H_2_O, 0.01 g CaCl_2_.6H_2_O, 2 g NaHCO_3_, 0.5 g l-cystine HCl, 2 ml Tween 80, 10 µl vitamin K1, 0.05 g haemin, 0.05 g bile salts, 11 g l-monosodium glutamate, 2.2 g tryptophan, and 4 ml resazurin (pH7) were added into 1 l of deionized water. A volume of 45 ml of medium was placed into glass jars and autoclaved at 121°C for 15 min.

#### pH-controlled, stirred batch culture fermentation

For each donor one independent batch culture was run. For each, batch culture vessels (4 × 100 ml) were set up and 45 ml of basal nutrient media were aseptically poured into each vessel. This system was left overnight with oxygen free nitrogen pumping through the medium at a rate of 15 ml/min with constant agitation and this continued throughout the course of fermentation. Before adding the faecal slurry, a water bath was used to set the temperature of the basal medium at 37°C, and a pH of between 5.4 and 5.6 was maintained throughout the course of fermentation using a pH meter (Electrolab pH controller, Tewksbury, UK) via the addition of 0.5 mol/l HCl or 0.5 mol/l NaOH. Stirring of faecal samples was maintained using a magnetic stirrer. For each donor three different substrates were prepared with one vessel containing each of the following substrates (1% w/v): OF, 2′FL, and OF + 2′FL (50/50 w/w). One vessel was set up as the negative control with no added carbohydrate. All vessels were inoculated with 5 ml of a 10% (w/v) faecal slurry (diluted with PBS). A sample (4 ml) was removed from each vessel after 0 h, 10 h , 24 h, and 48 h incubation to ensure enough sample was taken for bacterial, organic acid, and neurotransmitter analysis by fluorescence *in situ* hybridization-flow cytometry (FISH-FLOW), gas chromatography-flame ionization detection (GC-FID), and triple quadruple liquid chromatography–mass spectrometry (LC–MS QQQ), respectively.

### Enumeration of faecal microbial populations by flow cytometry fluorescence *in situ* hybridization (FISH-FLOW)

A 750 µl sample of batch culture fermentation effluent was centrifuged at 11 337 × *g* for 5 min. The supernatant was then discarded, and the pellet was then suspended in 375 µl filtered 0.1 mol/l, pH 7.4 PBS solution. Filtered 4% paraformaldehyde (PFA) at 4°C (1125 µl) were added and samples were stored at 4°C for 4 h. Samples were then washed thoroughly with PBS three times to remove PFA and resuspended in 150 µl PBS and 150 µl 99% ethanol. Samples were then stored at −20°C, until FISH analysis by flow cytometry could be conducted. The probes used in this study are presented in Table 1.

**Table 1.** Name, sequence, and target group of oligonucleotide probe used in this study for FISH of bacterial enumeration.

Probe	Sequence (5′–3′)	Targeted groups	References
Non Eub	ACTCCTACGGGAGGCAGC	Control probe complementary to EUB338	Wallner et al. (1993)
Eub338 I	GCTGCCTCCCGTAGGAGT	Most bacteria	Amann et al. (1990)
Eub338 I–II	GCAGCCACCCGTAGGTGT	*Planctomycetales*	Daims et al. (1999)
Eub338 I–II–III	GCTGCCACCCGTAGGTGT	*Verrucomicrobiales*	Daims et al. (1999)
Bif164	CATCCGGCATTACCACCC	*Bifidobacterium* spp.	Langendijk et al. (1995)
Lab158	GGTATTAGCAYCTGTTTCCA	*Lactobacillus* and *Enterococcus*	Franks et al. (1998)
Bac303	CCAATGTGGGGGACCTT	Most *Bacteroidaceae* and *Prevotellaceae*, some *Porphyromonadaceae*	Manz et al. (1996)

A volume of 75 µl of fixed samples were mixed with 500 µl filtered cold (4°C) 0.1 mol/l, pH 7.4 PBS and then centrifuged at 11 337 × *g* for 3 min. The resulting supernatant was then discarded, and pellets resuspended in 100 µl of TE-FISH (Tris/HCl 1 mol/l pH 8, EDTA 0.5 M pH 8, and filtered distilled water with the ratio of 1:1:8) containing lysozyme solution (1 mg/ml of 50 000 U/mg protein). Samples were then incubated for 10 min in the dark at room temperature and centrifuged at 11 337 × *g* for 3 min. Supernatants were discarded, and pellets washed with 500 µl filtered cold PBS by aspiration to disperse the pellet. Samples were then centrifuged at 11 337 × *g* for 3 min and supernatants discarded.

Pellets were resuspended in 150 µl of hybridization buffer, aspirated using a pipette and gently vortexed. Samples were centrifuged at 11 337 × *g* for 3 min and supernatants discarded. Pellets were resuspended in 1 ml of hybridization buffer. Aliquots (50 µl) of samples were placed in labelled 1.5 ml Eppendorf tubes and 4 µl of specific probes (50 ng/µl) were added. Samples were incubated at 35°C for at least 10 h in the dark.

Following incubation, 125 µl of hybridization buffer were added to each tube and gently vortexed. Samples were then centrifuged at 11 337 × *g* for 3 min and supernatants were discarded. Pellets were then washed with 175 µl of washing buffer solution and gently vortexed. Samples were incubated at 37°C for 20 min and centrifuged at 11 337 × *g* for 3 min. Supernatants were discarded and different volumes of filtered cold PBS (300, 600, and 1200 µl) were added based on flow cytometry load. Samples were kept at 4°C in the dark until flow cytometry measurements could be conducted. Fluorescence measures were performed on an BD Accuri™ C6 Plus (BD, Erembodegem, Brussels) measuring at 488 and 640 nm. A threshold of 9000 in forward scatter (FSC-A) and 3000 in side scatter (SSC-A) was placed to discard background noise, a gated area was applied in the main density dot to include 90% of the events. Flow rate was 35 ul/min, limit of collection was set for 100 000 events and analysed with Accuri Cflow Sampler software. Bacterial counts were then calculated through consideration of flow cytometry reading and PBS dilution.

### Organic acids by gas chromatography-flame ionization detection (GC-FID)

Samples (1.5 ml) of batch culture fluid were collected and centrifuged at 11 337 × *g* for 10 min. Supernatants were transferred to 1.5 ml Eppendorf tubes and stored at −80°C until analysis could be conducted. Sample extractions were performed according to (Richardson et al. 1989) with modifications. Briefly, 1 ml of sample was transferred into a labelled 100 mm ×16 mm glass tube (International Scientific Supplies Ltd, Bradford, UK) and 50 μl of 2-ethylbutyric acid (0.1 mol/l, internal standard), 500 µl concentrated HCl and 3 ml diethyl ether were added to each glass tube before vortexing for 1 min. Samples were centrifuged at 2000 × *g* for 10 min. The resulting diethyl ether (upper) layer of each sample was transferred to clean 100 ml screw top glass tubes. Ether extract (400 μl) and 50 μl *N*-tert-butyldimethylsilyl)-*N*-methyltrifluoroacetamide (MTBSTFA) were added into a GC screw-cap vial. Samples were left at room temperature for 72 h to allow samples to completely derivatise.

An Agilent/HP 6890 Gas Chromatograph (Hewlett Packard, UK) using an HP-5MS 30 m × 0.25 mm column with a 0.25 μm coating (cross-linked (5%-phenyl)-methylpolysiloxane, Hewlett Packard) was used for analysis of organic acids. Temperatures of injector and detector were 275°C, with the column temperature programmed from 63°C to 190°C at 15°C/min followed by 190°C for 3/min. Helium was the carrier gas (flow rate 1.7 ml/min; head pressure 133 KPa). A split ratio of 100:1 was used. Quantification of organic acids was achieved by calibration with acetic, propionic, butyric, lactate, and succinate acids in concentrations between 12.5 and 100 mmol/l. Mean metabolite concentrations were expressed as mmol/l.

### Neurotransmitter production by LC-MS QQQ

Samples (500 ul) of batch culture effluents were diluted to 1/100 and 1/1000 in LC-MS grade water and 1 ml was pipetted into HPLC screw top vials. For quantification of neurotransmitters a Shimadzu QQQ equipped with a Discovery HS F5 HPLC Column (3 mm particle size, L × I.D. 15 cm × 2.1 mm) maintained at 40°C was used. The mobile phase comprised of solvent A (0.1% v/v formic acid) and solvent B (acetonitrile containing 0.1% formic acid) at a flow rate of 0.25 ml/min. The gradient elution program was as follows: 2–5 min solvent B from 0% to 25%, 5–6 min solvent B from 25% to 95%, then holding for 2 min, 8–9 min from 95% to 0% and then until 15 min.

A LC/MS-8050 triple quadrupole (QQQ) detector was operated in the multiple reaction monitoring (MRM) mode using the polarity-switching electrospray ionization mode. Dry gas temperature was set at 200°C with a flow of 10 l/min. Injected sample volume was 4 µl. For the analysis of neurotransmitters, LC/MS Method Package for Primary Metabolites (Shimadzu Corporation, Kyoto, Japan) was used. The MRM transition for GABA was 104.10 > 87.05 m/z, tryptophan 205.10 > 188.15 m/z, serotonin 177.10 > 160.10 m/z, dopamine 154.10 > 91.05 m/z, kynurenic acid 190.10 > 144.10 m/z, norepinephrine 170.10 > 152.15 m/z, and epinephrine 184.10 > 166.10 m/z. Quantification of neurotransmitters was achieved via generation of a linear calibration curve ranging from 1.00 to 1000 ng/ml based on the detected signal proportional to concentration of the analyte. Good linearity of fit was considered as an *R*^2^ of greater than 0.99.

### Statistical analysis

Statistical Package for Social Science version 27 (SPSS Inc., Chicago, IL, USA) was used for all statistical analyses. Changes in bacteriology, neurotransmitter and organic acid production were analysed using a general linear model (GLM) to assess repeat measures. *Post hoc* comparisons were also performed in order to determine any significant differences between interventions at 48 h in organic acid and neurotransmitter production. All *post hoc* pairwise comparisons were corrected for type 1 errors using Bonferroni adjustment within each GLM. All tests were two tailed and *P*-values were considered significant at *P* ≤ .05 and are displayed by specified *P*-values. Graphs were generated in GraphPad Prism version 10.0.0 for Windows, GraphPad Software, San Diego, California, www.graphpad.com.

## Results

### Neurotransmitter production

**Figure 1.**
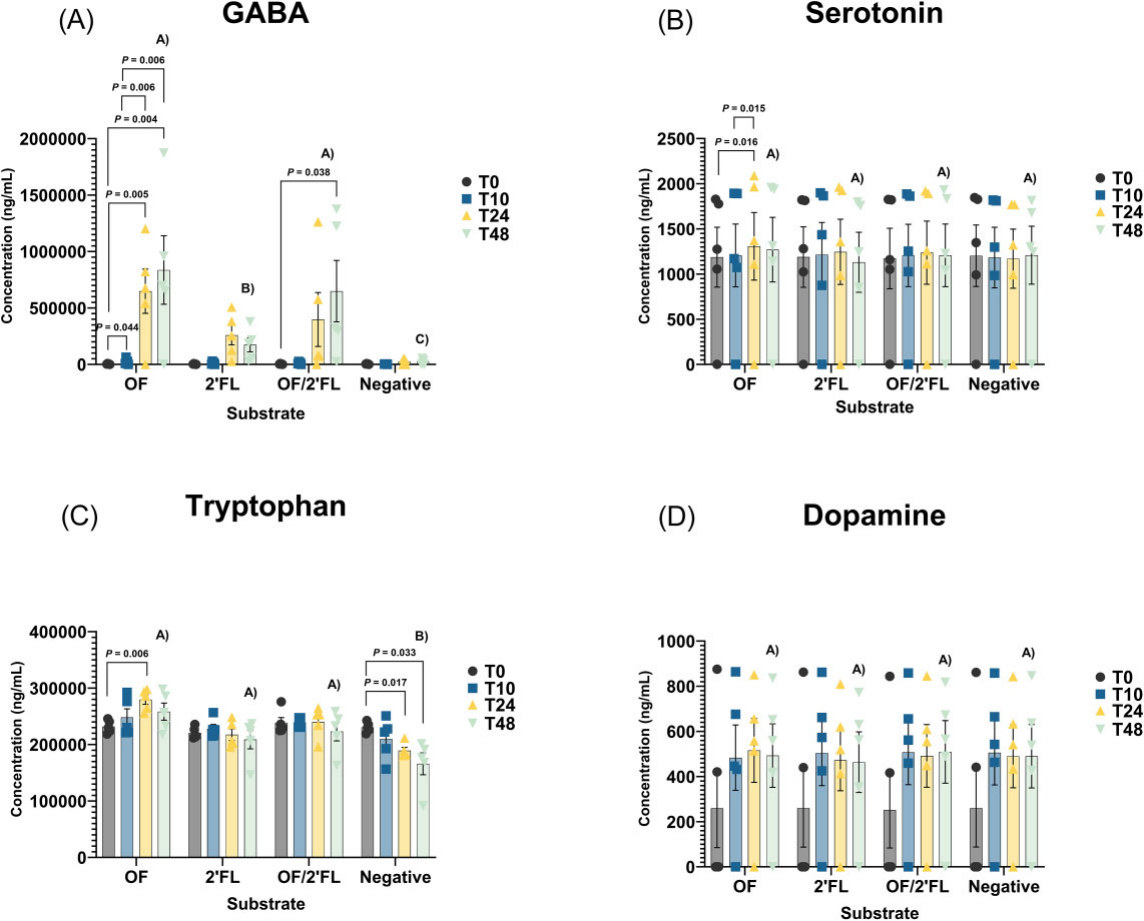
LC-MS analysis of neurotransmitter concentrations—(A) GABA, (B) serotonin, (C) tryptophan, and (D) dopamine in the supernatant of effluents collected from vessel 1–4 at 0, 10, 24, and 48 h representing mean (*n* = 5) and standard error (SE) of the data with individual volunteer data points. Concentrations reported in (ng/ml). Results that are statistically significant within respective treatments are displayed by specified *P-*values. Significant differences between treatments at 48 h are indicated by differing letters (*P* ≤ .05). Abbreviations: OF = oligofructose; 2′FL = 2′fucosyllactose.

Figure 1 report the concentrations of GABA, serotonin, tryptophan, and dopamine over the course of the 48 h fermentation. Norepinephrine, epinephrine, and kynurenic acid were below the limit of detection and were therefore excluded from analysis. Mean and specific donor data are presented in Tables S1 and S2 (Supporting Information).

The largest changes seen in neurotransmitter production across all substrates tested were recorded for GABA. The extent of change and fermentation patterns varied substantially across the substrates tested. Of all substrates tested, OF induced the largest increases in GABA at 48 h going from 3605.01 ± 1347.35 standard error (SE) ng/ml to 836 187.28 ± 303 310 (SE) ng/ml (832 582.27 mean difference; *P* = .004). In contrast, increases in GABA concentration in the 2′FL fermentations were highly heterogenous amongst donors, subsequently inducing the smallest average increase at 25,5763.59 ng/ml ± 84 953 (SE) above baseline at 24 h (Fig. 1A; Table S1, Supporting Information).

Figure 1(B) and (C) report the data for both serotonin and tryptophan. Both tryptophan and serotonin concentrations remained virtually unchanged throughout the course of fermentation. The only significant increase in tryptophan production was detected in the OF treatment vessel at 24 h fermentation (*P* = .004). Similarly, the only significant increases in serotonin production were detected on OF 0–24 h (*P* = .016) and 10–24 h (*P* = .015) fermentation (Table S1, Supporting Information).

Finally, no significant differences were detected in dopamine concentrations throughout the entire 48 h fermentation across any of the substrates tested (Fig. 1D; Table S1, Supporting Information).

### Organic acids

**Figure 2.**
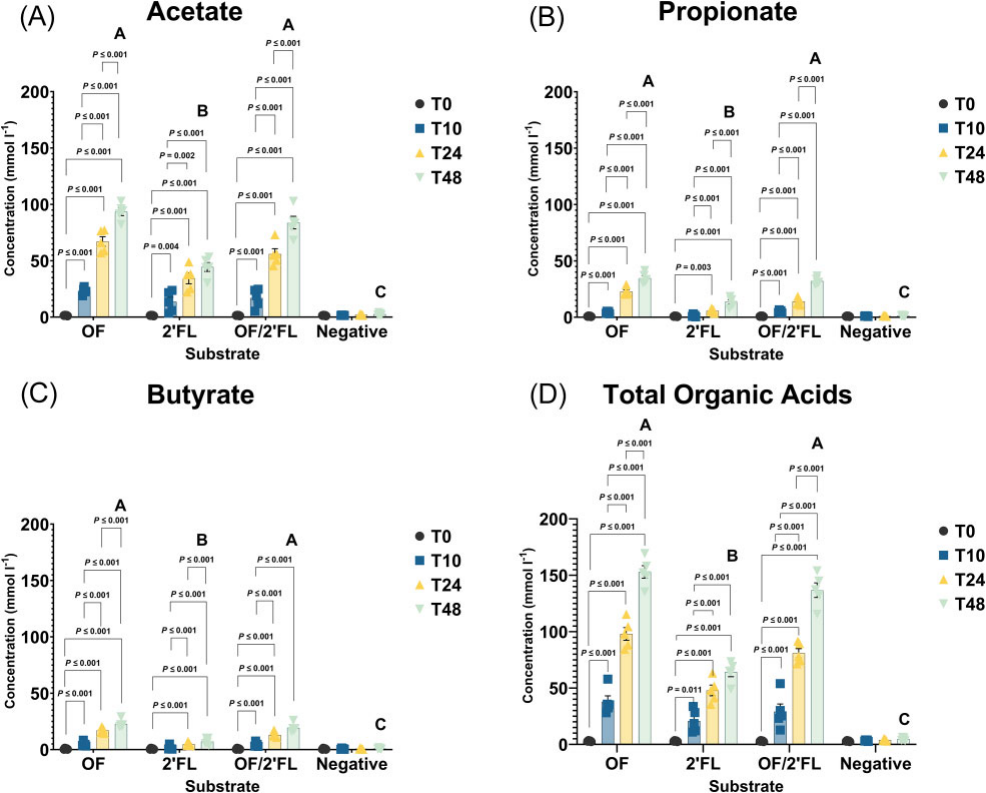
GC-FID analysis of organic acid concentrations—(A) acetate, (B) propionate, (C) butyrate, and (D) total organic acids in the supernatant of effluents collected from vessel 1–4 at 0, 10, 24, and 48 h representing (*n* = 5) of the data (all points). Concentration reported in (mmol/I) mean and SE. Results that are statistically significant within respective treatments are displayed by specified *P-*values. Significant differences between treatments at 48 h are indicated by differing letters (*P* ≤ .05). Abbreviations: OF = oligofructose; 2′FL = 2′fucosyllactose.

Figure 2(A)–(D) report concentrations for acetate, propionate, butyrate, and total organic acids throughout the course of fermentation. Lactate and succinate concentrations along with mean and specific donor data for all organic acids are reported in Tables S3 and S4 (Supporting Information).

Acetate was the most abundant organic acid detected representing between 53.9% and 69.2% of total organic acids produced at the end of fermentation. Acetate concentrations were highest in all treatments tested at 48 h and were all statistically significant compared with 0 h (Fig. 2A; Table S3, Supporting Information). Increases in acetate production varied substantially between treatments with OF producing the largest acetogenic effect, averaging an increase of 91.94 ± 3.41 (SE) mmol/I above baseline. Lowest increases in acetate production were recorded on 2′FL averaging an increase of 42.98 ± 3.94 (SE) mmol/I above baseline. Increases in acetate in both OF and OF/2′FL treatments being statistically greater than sole 2′FL at 48 h (both *P* ≤ .001) (Table S3, Supporting Information).

Propionate production accounted for between 18.30% and 26.7% of total organic acids with all substrates inducing significant increases in propionate at 48 h compared with 0 h (Fig. 2B). Most notable propiogenic substrate was OF, averaging an 33.82 ± 1.99 (SE) mmol/I increase above 0 h. The combination of OF/2′FL produced similar results inducing an average 31.21 ± 1.34 (SE) mmol/I increase above 0 h sampling (Table S3, Supporting Information). Lowest increases in propionate were seen in the 2′FL treatment vessel at just 10.85 ± 1.66 (SE) mmol/I above baseline. The increases in propionate recorded by both OF and OF/2′FL combination being statistically different from sole 2′FL at 48 h (both *P* ≤ .001) (Fig. 2B; Table S4, Supporting Information).

All substrates tested resulted in significant increases in butyrate production, yet substantial differences in butyrate production were seen between substrates. Most noticeable butyrogenic substrates were OF and OF/2′FL with an average 22.22 ± 1.44 (SE) mmol/I and 18.65 ± 1.26 (SE) mmol/I increase above respective baseline samples. The increases seen in butyrate production in both OF and OF/2′FL treatments were statistically greater compared to 2′FL alone at 48 h (both *P* ≤ .001) (Fig. 2C; Table S3, Supporting Information).

With respect to lactate, all substrates resulted in significant increases in lactate at 10 h fermentation mark (Table S3, Supporting Information). Thereafter, lactate concentrations declined in all substrates. No significant differences were detected between substrates at 48 h (Table S3, Supporting Information).

All substrates tested resulted in significant increases in succinate (Table S3, Supporting Information). Yet, increases in succinate production varied substantially between substrates with OF and combination of OF/2′FL inducing significant increases in succinate production at 10 h (OF *P* ≤ .001) and (OF/2′FL *P* = .003), 24 h (both *P* ≤ .001) and 48 h (both *P* ≤ .001), respectively (Table S3, Supporting Information). In contrast, significant increases in succinate production in the 2′FL treatment vessel were only detected at 48 h (*P* = .003). Furthermore, the increases detected in succinate concentrations at 48 h fermentation in both the OF and OF/2′FL treatment vessels were statistically different from 2′FL alone (both *P* ≤ .001) (Table S3, Supporting Information).

Finally, regarding total organic acids, all substrates resulted in significant increases in total organic acids at the end of fermentation. OF induced the largest increases at 150.018 ± 5.38 (SE) mmol/I at 48 h and was statistically greater compared to 2′FL (*P* ≤ .001), but not OF/2′FL. Smallest increases in total organic acids were recorded on 2′FL at 61.24 ± 4.02 (SE) mmol/I above 0 h (Fig. 2D; Table S3, Supporting Information).

### Bacterial enumeration

**Figure 3.**
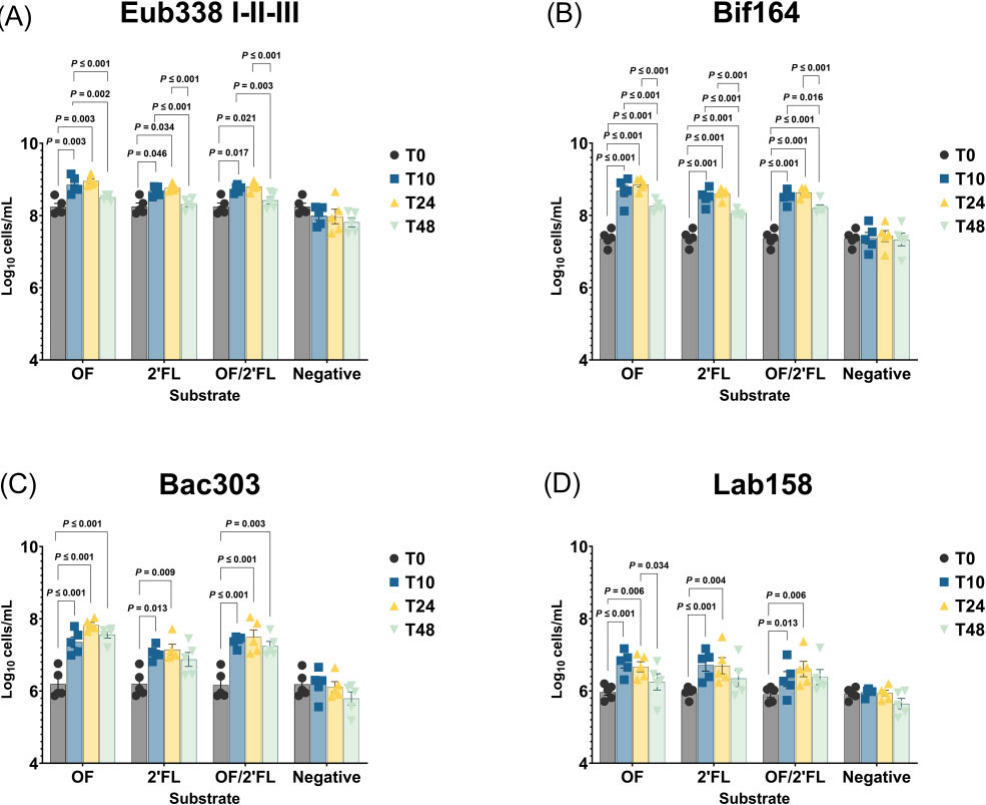
Bacterial groups measured by FISH-FLOW (Log_10_ cells/ml) using probes: (A) Total bacteria (Eub338 I–II–III), (B) *Bifidobacterium* spp. (Bif164), (C) Most *Bacteroidaceae* and *Prevotellaceae*, some *Porphyromonadaceae* (Bac303), and (D) *Lactobacillus/Enterococcus* (Lab158) at 0, 10, 24, and 48 h. Mean and SE (all data points; *n* = 5). Results that are statistically significant within respective treatments are displayed by specified *P*-values. Abbreviations: OF = oligofructose; 2′FL = 2′fucosyllactose.

In order to determine shifts in bacterial populations, four 16S-rRNA fluorescence *in situ* hybridization probes were used to identify changes in numbers of total bacteria and three specifically targeted microbial groups. Results of significant bacterial group counts during the batch culture fermentation are shown in Fig. 3(A)–(D). Mean and donor specific data is reported in Tables S5 and S6 (Supporting Information).

Significant increases in total bacterial counts (Eub338-I, II, and III) were observed across all substrates with largest increases in microbial loads peaking at 24 h (Fig. 3A). OF induced the largest increases in total bacteria counts averaging 0.72 log_10_ ± 0.14 cells/ml above 0 h (*P* ≤ .001). Smallest increases in total bacterial counts were induced on 2′FL and OF/2′FL combination at 0.52 log_10_ ± 0.12 cells/ml and 0.55 log_10_ ± 0.13 cells/ml above 0 h. Both of these were statistically significant from respective baseline samples (Fig   3A; Table S5, Supporting Information).

Largest changes in microbial numbers were recorded in *Bifidobacterium* (Bif164) counts with all three substrates tested recording significant increases across the course of fermentation. Yet, increases in Bif164 counts varied between the substrates tested with OF inducing the largest average increases in Bif164 counts at 1.49 log_10_ ± 0.13 cells/ml (SE) (*P* ≤ .001) at 24 h. Both 2′FL and the combination of OF/2′FL inducing similar increases of Bif164 counts at 1.22 log_10_ ± 0.11 and 1.26 log_10_ ± 0.11 (SE) cells/ml (Fig. 3B; Table S5, Supporting Information). The changes seen in Bif164 counts correlating with those documented in acetate and GABA production throughout the course of fermentation.

All substrates resulted in significant increases in Bac303 counts. Significant differences were detected amongst treatments, with OF inducing largest increases in Bac303 at 24 h at 1.64 log_10_ ± 0.23 cells/ml (SE) and remained significant until the end of fermentation (all *P* ≤ .001). In contrast, 2′FL induced the smallest increases in Bac303 counts at 0.99 log_10_ ± 0.25 cells/ml (SE), both 8 h and 24 h samples recording significant differences compared to 0 h (Fig. 3C; Table S5, Supporting Information).

Lastly, significant differences in Lab158 counts were detected across all substrates tested (Fig. 3D). Largest changes in Lab158 counts were recorded on OF with 0.8 log_10_ ± 0.14 cells/ml (SE) increase at 8 h (*P* ≤ .001), sole 2′FL recording similar average increases at 0.76 log_10_ ± 0.17 cells/ml (SE) (*P* ≤ .001). Smallest average increases in Lab158 counts were in the OF/2′FL treatment vessel at just 0.42 log_10_ ± 0.21 cells/ml (SE) at 24 h fermentation (Fig. 3D; Table S5, Supporting Information). Interestingly, within both the sole 2′FL and OF/2′FL combination fermentations several donors recorded higher average increases in Lab158 counts at 24 h fermentation, each of which were maintained until the end of fermentation (Table S6, Supporting Information).

## Discussion

In this *in vitro* batch culture investigation, we aimed to assess if the prebiotic OF and prebiotic candidate 2′FL, alone and in combination, could induce physiologically relevant changes in neuroactive metabolites and organic acid production.

Largest increases in neurotransmitters were seen in GABA production and were highest in OF and OF/2′FL fermentations peaking at 48 h, whereas smaller increases were detected on 2′FL peaking at 24 h, respectively, results being highly heterogenous amongst individual donors (Fig. 1; Tables S1 and S2, Supporting Information). Taking this into consideration it has been recently documented that rates of GABA synthesis can be dramatically influenced by the type of hexose and pentose sugars used during fermentation (Strandwitz et al. 2019, Cataldo et al. 2020).

Within the gut several microorganisms including *Bifidobacterium, Bacteroides*, and *Lactobacillus* have developed several mechanisms for producing GABA. GABA can be produced via the decarboxylation of l-glutamate or via the conversion of arginine to provide a protective mechanism on the low pH of the intestinal environment (Strandwitz et al. 2019, Otaru et al. 2021). We did not add arginine to our basal media thus the majority of GABA production likely occurred via the decarboxylation of l-glutamate.

Other mechanisms by which GABA production can occur include utilization of acetate and lactate as intermediates in the citric acid cycle (Rowlands et al. 2017). Consequently, given that there were significant increases in both acetate and lactate seen throughout the course of fermentation, specifically on OF and OF/2′FL, the additional increases seen in GABA in this study may be from utilization of both acetate and lactate via the gut microbiota. This coincides with results documented by (Frost et al. 2014) who noted that ^13^C-labelled acetate was able to cross the blood–brain barrier regulating GABA production in the hypothalamus. This suggests that increasing GABA production via increasing acetate production through the use of the prebiotics may provide a means of increasing GABA production. This is of particular importance given GABA concentrations often correlate with levels of depression and several other mood state disorders (Brady et al. 2013, Al-Khafaji et al. 2020).

Small increases in both tryptophan and serotonin production were detected only on OF fermentations at 24 h (Fig. 1B and C; Table S1, Supporting Information). While much remains unknown about the ability of the gut microbiota to produce tryptophan, dietary tryptophan can be utilized by the gut microbiota to produce several derivative metabolites including indole and serotonin as well as entering the kynurenine pathway resulting in the production of kynurenic, quinolinic, or picolinic acid (Kaur et al. 2019, Gao et al. 2020). Yet, in this study, kynurenic acid was under the limit of detection and the increases recorded in both serotonin and tryptophan production were relatively low compared to the increases seen in GABA production and likely do not reflect those seen *in vivo*. Taking this into consideration the conversion of tryptophan to serotonin occurs in the presence of enterochromaffin cells, serotonergic neurons and necessary cofactors including pyridoxine-5-phosphate (Reigstad et al. 2015). Furthermore, while the pH used this study reflected the pH of the proximal colon, allowing for adequate exploration of GABA production by the microbiota, it likely did not provide optimal conditions for production of metabolites at the higher pH within the transverse and distal region of the colon. Therefore, it would be beneficial for future work to model proximal, transverse, and distal regions of the colon combined with the addition of all necessary precursors and cofactors in order to more adequately explore neurotransmitter production.

Concentrations of acetate, propionate, butyrate, lactate, and succinate all increased across the course of fermentation with OF and OF/2′FL in combination recording significantly greater increases compared to 2′FL (Fig. 2; Tables S3 and S4, Supporting Information).

Metabolically derived organic acids are speculated to play a pivotal role in context of the gut–brain axis being linked to several health benefits. For example acetate, which was significantly increased in the OF and to a lesser extent OF/2′FL fermentations, has been linked to reductions in inflammation by regulating the expression of proinflammatory cytokines IL-6, TNF-α, and IL-1β (Soliman et al. 2012, Underwood et al. 2015). Propionate is speculated to play a key role in regulating blood brain barrier permeability, along with protecting against lipopolysaccharide-mediated blood–brain barrier disruption (Braniste et al. 2014, Hoyles et al. 2018). Butyrate, again significantly enhanced in OF and OF/2′FL fermentations, has been shown to aid in regulating the expression of GABA receptors, enterochromaffin cells, brain-derived neurotrophic factor and glial-derived neurotrophic factor in mice and rats (Moris and Vega 2003, Intlekofer et al. 2013, Savignac et al. 2013, Reigstad et al. 2015) as well as being documented to decrease histone acetylation in piglets (Kien et al. 2008).

Organics acids, specifically acetate has been linked to improvements in satiety regulation via stimulation of leptin production in adipocytes (Zaibi et al. 2010), while succinate and propionate can act as precursors to gluconeogenesis (den Besten et al. 2013, Soty et al. 2015).

Significant increases in *Bifidobacterium* spp., *Bacteroides*/*Prevotella*, and *Lactobacillus*/*Enterococcus were* detected across all substrates with largest increases being documented in the OF fermentation (Fig. 3A–D). In contrast, a high degree of heterogeneity was detected amongst donors in response to 2′FL, except when in the presence of OF where this heterogeneity appeared to be mitigated.

The response of the gut microbiota, in particular *Bifidobacterium* spp., to OF is well-characterized with its effect being demonstrated in both *in vitro* (Wang and Gibson 1993, Pompei et al. 2008) and *in vivo* studies (Kolida et al. 2007, Vandeputte et al. 2017). The ability of the healthy adult gut microbiota to utilize 2′FL is extremely limited and our results are in line with those documented by (Yu et al. 2013, Suligoj et al. 2020, Ryan et al. 2021), indicating that a large responder/nonresponder status exists to 2′FL supplementation.

Taking this into consideration, the ability of the microbiota to utilize specific HMOs is documented to be highly species and even strain specific (Lawson et al. 2020, Sakanaka et al. 2020, Jackson et al. 2022). This indicates that, if an individual is to utilize HMOs, they must possess the necessary microorganisms prior to supplementation. For example, within the 2′FL fermentations several donors documented noticeable increases in Lab158 counts compared to OF fermentation (Fig. 3D; Tables S5 and S6, Supporting Information). This is interesting given that it has been previously documented that *Lactobacillus* are not readily able to utilize intact HMOs to any real extent (Jackson et al. 2022). As there were also notable increases in *Bifidobacterium* within these individuals it is likely that increases in *Lactobacillus* occurred as result on scavenging on both lactose and fucose from the extracellular degradation of HMOs by bifidobacteria (Zuniga et al. 2018). This further adds to the evidence that the mutualistic behaviour existing between microorganisms found within the gut is a critical character in helping to shape a diverse and flexible ecosystem (Jackson et al. 2022).

Our study is not, however, without limitation. It should be acknowledged that the use of *in vitro* fermentation models to identify changes in microbial composition and resulting metabolites does not necessary capture changes seen *in vivo*. Furthermore, it is important to note that while we did see significant increases in the production of several neurotransmitters evidence supporting the ability of gut-derived neurotransmitters to cross the blood–brain barrier is not well-established (Strandwitz 2018) and findings should be interpreted as such. However, *in vitro* models do allow for the testing of novel substrate combinations as prescreening tools for assessing potential changes in microbial composition and metabolite production prior to conducting human intervention trials and minimizing the heterogeneity seen *in vivo*.

## Conclusion

Overall, the results of study imply that OF and combinations of OF/2′FL were able to generate physiologically relevant increases in GABA and organic acids, all of which were maintained until the end of fermentation (all *P* ≤ .05), along with noticeable increases in *Bifidobacterium, Bacteroides*, and *Lactobacillus* counts. Whereas the ability of 2′FL to induce physiologically relevant increases in GABA and organic acid production along with substantiated changes in bacterial numbers appears to be highly donor specific, expect for when combined with OF, suggesting a strong responder/nonresponder status exists. Additionally, OF was able to stimulate small increases in both serotonin and tryptophan, suggesting bacterial production of neuroactive metabolites may occur in the absence of colonic cells. However, these concentrations were relatively low compared to GABA indicating that the gut microbiota is not the primary production pathway and that the presence of the necessary enteroendocrine cells is required. Overall, these results suggest that the prebiotic OF and combination of OF/2′FL should be taken forward to a human intervention trial to determine their *in vivo* effects.

## Supplementary Material

fiad100_Supplemental_File

## Data Availability

The data will be made available from the corresponding upon reasonable request.
