# Supplementary material for: Oligofructose alone and in combination with 2′fucosyllactose induces physiologically relevant changes in γ-aminobutyric acid and organic acid production compared to sole 2′fucosyllactose supplementation: an in vitro study
Source: FEMS Microbiol Ecol. 2023 Aug 31;99(9):fiad100. doi: 10.1093/femsec/fiad100 (PMC10481994; doi:10.1093/femsec/fiad100)
Supplement: fiad100_Supplemental_File [file fiad100_supplemental_file.docx]

## **Supplemental Data Table 1 Mean neurotransmitter concentrations using pH-controlled *in vitro* batch culture fermentation at 0, 10, 24, and 48 h.**

| **GABA** | **T0** | **T10** | | **T24** | | **T48** | |
| --- | --- | --- | --- | --- | --- | --- | --- |
|  | **Mean (SE)** | **Mean (SE)** | ***P* (a)** | **Mean (SE)** | ***P* (a)** | **Mean (SE)** | ***P* (a)** |
| **OF** | 3605.01 (1347.34) | 22521.68 (11 129.67) | **0.04** | 648 363.68 (196 093.75) | **0.01** | 836 187.28 (303 309.74) | **0.006a** |
| **2'FL** | 3442.02 (1215.50) | 10 600.01 (54 38.81) | 1.00 | 259 205.61 (86167.27) | 0.78 | 173 619.59 (62499.15) | 1.00b |
| **OF/2'FL** | 3620.14 (1330.43) | 11 033.90 (4680.11) | 1.00 | 398 177.21 (239 084.58) | 0.15 | 648 721.89 (271 926.65) | **0.04a** |
| **Negative** | 3616.91 (1128.03) | 2684.10 (765.47) | 1.00 | 24 783.72 (10 623.96) | 1.00 | 22 208.95 (10 595.85) | 1.00c |
| ***P* (b)** | 1.00 | 0.25 | | 0.09 | | **0.05** | |

| **Serotonin** | **T0** | **T10** | | **T24** | | **T48** | |
| --- | --- | --- | --- | --- | --- | --- | --- |
|  | **Mean (SE)** | **Mean (SE)** | ***P* (a)** | **Mean (SE)** | ***P* (a)** | **Mean (SE)** | ***P* (a)** |
| **OF** | 1187.13 (331.21) | 1205.84 (347.58) | 1.00 | 1307.19 (374.12) | **0.02** | 1269.80 (357.09) | 0.39a |
| **2'FL** | 1188.22 (334.62) | 1215.08 (355.87) | 1.00 | 1246.12 (361.25) | 0.64 | 1130.60 (332.18) | 1.00a |
| **OF/2'FL** | 1172.09 (334.56) | 1205.94 (345.19) | 1.00 | 1236.77 (349.36) | 0.44 | 1207.17 (346.64) | 1.00a |
| **Negative** | 1202.60 (340.33) | 1182.57 (335.46) | 1.00 | 1170.84 (327.32) | 1.00 | 1208.59 (320.75) | 1.00a |
| ***P* (b)** | 1.00 | 1.00 | | 0.99 | | 0.99 | |

LC-MS analysis of GABA and serotonin concentrations in the supernatant of effluents collected from vessel 1-4 at 0, 10 24 and 48 h representing the mean (*n* = 5) and standard error (SE) of the data. Concentration reported in (ng/mL). **(a)** Significant differences compared with respective 0 h sampling are identified with specified *P* values (grey columns). **(b)** Significant differences between substrates at 0, 10, 24 and 48 h are indicated by specified *P* values (orange column). Significant differences between substrates at 48h are indicated by differing letters (grey column). **Abbreviations:** OF = oligofructose; 2’FL = 2’fucosyllactose

| **Dopamine** | **T0** | **T10** | | **T24** | | **T48** | |
| --- | --- | --- | --- | --- | --- | --- | --- |
|  | **Mean (SE)** | **Mean (SE)** | ***P* (a)** | **Mean (SE)** | ***P* (a)** | **Mean (SE)** | ***P* (a)** |
| **OF** | 259.20 (174.26) | 483.47 (144.86) | 0.88 | 515.67 (141.38) | 0.57 | 492.78 (140.66) | 0.39a |
| **2'FL** | 260.44 (172.92) | 504.36 (144.53) | 0.7 | 472.31 (134.94) | 0.98 | 463.51 (134.07) | 1.00a |
| **OF/2'FL** | 252.00 (168.45) | 506.93 (142.81) | 0.61 | 491.46 (138.97) | 0.71 | 509.03 (138.64) | 1.00a |
| **Negative** | 260.51 (172.80) | 505.69 (142.90) | 0.69 | 490.17 (139.80) | 0.79 | 489.82 (140.48) | 1.00a |
| ***P* (b)** | 1.00 | 0.99 | | 0.98 | | 0.97 | |

| **Tryptophan** | **T0** | **T10** | | **T24** | | **T48** | |
| --- | --- | --- | --- | --- | --- | --- | --- |
|  | **Mean (SE)** | **Mean (SE)** | ***P* (a)** | **Mean (SE)** | ***P* (a)** | **Mean (SE)** | ***P* (a)** |
| **OF** | 231 829.66 (5043.73) | 248 312.00 (15012.54) | 1.00 | 279 613.74 (8319.84) | **0.01** | 258 392.15 (15 163.45) | 0.39a |
| **2'FL** | 220 519.04 (4664.84) | 228 039.90 (7593.53) | 1.00 | 217 513.88 (10 389.75) | 1.00 | 208 840.54 (16 385.77) | 1.00a |
| **OF/2'FL** | 238 282.03 (9598.23) | 239 076.92 (3742.07) | 1.00 | 240 143.72 (12 042.85) | 1.00 | 223 110.55 (16 564.48) | 1.00a |
| **Negative** | 230 615.23 (4034.57) | 209 996.53 (16 263.66) | 0.57 | 189 245.02 (5742.87) | 0.02 | 165 878.40 (19 420.11) | 1.00b |
| ***P* (b)** | 1.00 | 0.165 | | **≤ 0.001** | | 0.012 | |

LC-MS analysis of dopamine and tryptophan concentrations in the supernatant of effluents collected from vessel 1-4 at 0, 10 24 and 48 h representing the mean (*n* = 5) and standard error (SE) of the data. Concentration reported in (ng/mL). **(a)** Significant differences compared with respective 0 h sampling are identified with specified *P* values (grey columns). **(b)** Significant differences between substrates at 0, 10, 24 and 48 h are indicated by specified *P* values (orange row). Significant differences between substrates at 48h are indicated by differing letters (grey column). **Abbreviations:** OF = oligofructose; 2’FL = 2’fucosyllactose

## **Supplemental Data Table 2 Individual neurotransmitter concentrations using pH-controlled *in vitro* batch culture fermentation at 0, 10, 24, and 48 h.**

| GABA |  | T 0 | T 10 | T 24 | T 48 |
| --- | --- | --- | --- | --- | --- |
| OF | D1 | 0.00 | 0.00 | 0.00 | 0.00 |
|  | D2 | 8321.51 | 26 452.49 | 120 1822.56 | 1 871 902.65 |
|  | D3 | 2582.76 | 63 631.19 | 822 092.78 | 958 973.30 |
|  | D4 | 3651.03 | 13 626.42 | 676 310.88 | 697 401.34 |
|  | D5 | 3469.78 | 8898.30 | 541 592.22 | 652 659.12 |
| 2'FL | D1 | 226 870.90 | 256 768.12 | 195 666.71 | 206 978.00 |
|  | D2 | 213 229.43 | 226 184.19 | 248 452.11 | 224 037.82 |
|  | D3 | 211 748.14 | 215 851.22 | 235 932.38 | 236 802.94 |
|  | D4 | 235 805.84 | 226 533.55 | 201 910.08 | 146 354.07 |
|  | D5 | 214 940.89 | 214 862.43 | 205 608.10 | 230 029.89 |
| OF/2'FL | D1 | 0.00 | 0.00 | 0.00 | 23 631.03 |
|  | D2 | 8225.82 | 26 150.80 | 575 892.04 | 1 374 958.14 |
|  | D3 | 2586.21 | 6501.57 | 80 218.58 | 298 903.10 |
|  | D4 | 3563.56 | 17 053.34 | 126 1969.41 | 1 224 407.69 |
|  | D5 | 3725.12 | 5463.82 | 72 806.03 | 321 709.53 |
| Negative | D1 | 235 554.80 | 250 838.50 | 211 434.81 | 201 325.51 |
|  | D2 | 229 958.87 | 227 712.55 | 189 234.79 | 176 458.16 |
|  | D3 | 242 622.28 | 156 486.47 | 183 276.24 | 91 655.06 |
|  | D4 | 225 924.82 | 222 171.96 | 180 821.41 | 191 547.31 |
|  | D5 | 219 015.38 | 192 773.18 | 181 457.88 | 168 405.98 |

Individual volunteer GABA concentrations across 0, 10, 24, and 48 h fermentation. Concentration in (ng/mL). **Abbreviations:** OF = oligofructose; 2’FL = 2’fucosyllactose

| Serotonin |  | T 0 | T 10 | T 24 | T 48 |
| --- | --- | --- | --- | --- | --- |
| OF | D1 | 1828.543 | 1891.399 | 1967.226 | 1938.207 |
|  | D2 | 1777.178 | 1894.009 | 2089.146 | 1957.425 |
|  | D3 | 1054.951 | 1073.992 | 1108.638 | 1141.57 |
|  | D4 | 1274.958 | 1169.802 | 1370.916 | 1311.815 |
|  | D5 | 0 | 0 | 0 | 0 |
| 2'FL | D1 | 1821.85 | 1866.099 | 1926.757 | 1757.88 |
|  | D2 | 1813.422 | 1899.155 | 1962.383 | 1797.386 |
|  | D3 | 1024.94 | 874.4826 | 984.1845 | 852.496 |
|  | D4 | 1280.865 | 1435.652 | 1357.258 | 1245.26 |
|  | D5 | 0 | 0 | 0 | 0 |
| OF/2'FL | D1 | 1821.92 | 1862.844 | 1892.053 | 1834.004 |
|  | D2 | 1826.322 | 1886.344 | 1919.713 | 1932.08 |
|  | D3 | 1052.394 | 1026.947 | 1113.632 | 1042.507 |
|  | D4 | 1159.836 | 1253.587 | 1258.467 | 1227.245 |
|  | D5 | 0 | 0 | 0 | 0 |
| Negative | D1 | 1827.556 | 1819.004 | 1768.697 | 1678.534 |
|  | D2 | 1846.128 | 1810.73 | 1770.456 | 1813.248 |
|  | D3 | 991.7268 | 984.6516 | 992.3396 | 1247.946 |
|  | D4 | 1347.575 | 1298.477 | 1322.696 | 1303.235 |
|  | D5 | 0 | 0 | 0 | 0 |

Individual volunteer serotonin concentrations across 0, 10, 24, and 48 h fermentation. Concentration in (ng/mL). **Abbreviations:** OF = oligofructose; 2’FL = 2’fucosyllactose

| **Tryptophan** |  | **T 0** | **T 10** | **T 24** | **T 48** |
| --- | --- | --- | --- | --- | --- |
| **OF** | D1 | 1828.543 | 1891.399 | 1967.226 | 1938.207 |
|  | D2 | 1777.178 | 1894.009 | 2089.146 | 1957.425 |
|  | D3 | 1054.951 | 1073.992 | 1108.638 | 1141.57 |
|  | D4 | 1274.958 | 1169.802 | 1370.916 | 1311.815 |
|  | D5 | 0 | 0 | 0 | 0 |
| **2'FL** | D1 | 1821.85 | 1866.099 | 1926.757 | 1757.88 |
|  | D2 | 1813.422 | 1899.155 | 1962.383 | 1797.386 |
|  | D3 | 1024.94 | 874.4826 | 984.1845 | 852.496 |
|  | D4 | 1280.865 | 1435.652 | 1357.258 | 1245.26 |
|  | D5 | 0 | 0 | 0 | 0 |
| **OF/2'FL** | D1 | 1821.92 | 1862.844 | 1892.053 | 1834.004 |
|  | D2 | 1826.322 | 1886.344 | 1919.713 | 1932.08 |
|  | D3 | 1052.394 | 1026.947 | 1113.632 | 1042.507 |
|  | D4 | 1159.836 | 1253.587 | 1258.467 | 1227.245 |
|  | D5 | 0 | 0 | 0 | 0 |
| **Negative** | D1 | 1827.556 | 1819.004 | 1768.697 | 1678.534 |
|  | D2 | 1846.128 | 1810.73 | 1770.456 | 1813.248 |
|  | D3 | 991.7268 | 984.6516 | 992.3396 | 1247.946 |
|  | D4 | 1347.575 | 1298.477 | 1322.696 | 1303.235 |
|  | D5 | 0 | 0 | 0 | 0 |

Individual volunteer tryptophan concentrations across 0, 10, 24, and 48 h fermentation. Concentration in (ng/mL). **Abbreviations:** OF = oligofructose; 2’FL = 2’fucosyllactose

| Dopamine |  | T 0 | T 10 | T 24 | T 48 |
| --- | --- | --- | --- | --- | --- |
| OF | D1 | 875.4 | 864.102 | 850.9363 | 836.0207 |
|  | D2 | 0 | 675.7037 | 655.9527 | 651.2717 |
|  | D3 | 0 | 445.85 | 557.827 | 558.683 |
|  | D4 | 0 | 0 | 0 | 0 |
|  | D5 | 420.6117 | 431.7067 | 513.6395 | 417.9245 |
| 2'FL | D1 | 862.447 | 861.6107 | 809.4443 | 772.3153 |
|  | D2 | 0 | 661.6107 | 619.8304 | 629.4703 |
|  | D3 | 0 | 573.3697 | 519.1633 | 561.9866 |
|  | D4 | 0 | 0 | 0 | 0 |
|  | D5 | 439.7643 | 425.2434 | 413.1043 | 353.7967 |
| OF/2'FL | D1 | 843.5437 | 858.521 | 845.238 | 817.4137 |
|  | D2 | 0 | 655.4917 | 608.374 | 672.3763 |
|  | D3 | 0 | 561.388 | 555.026 | 565.3753 |
|  | D4 | 0 | 0 | 0 | 0 |
|  | D5 | 416.4767 | 459.234 | 448.6927 | 489.9617 |
| Negative | D1 | 861.3027 | 858.1283 | 842.6837 | 846.2063 |
|  | D2 | 0 | 664.7643 | 633.3073 | 636.363 |
|  | D3 | 0 | 542.4397 | 541.599 | 538.9684 |
|  | D4 | 0 | 0 | 0 | 0 |
|  | D5 | 441.2233 | 463.1307 | 433.266 | 427.541 |

Individual volunteer dopamine concentrations across 0, 10, 24, and 48 h fermentation. Concentration in (ng/mL). **Abbreviations:** OF = oligofructose; 2’FL = 2’fucosyllactose

## **Supplemental Data Table 3 Mean organic acid concentrations using pH-controlled *in vitro* batch culture fermentation at 0, 10, 24, and 48 h.**

| **Acetate** | **Time (h)** | **T0** | **T10** | | **T24** | | **T48** | |
| --- | --- | --- | --- | --- | --- | --- | --- | --- |
|  |  | **Mean (SE)** | **Mean (SE)** | ***P* (a)** | **Mean (SE)** | ***P* (a)** | **Mean (SE)** | ***P* (a)** |
| **Substrate** | **OF** | 1.43 (0.10) | 23.16 (1.35) | **≤ 0.001** | 67.02 (4.28) | **≤ 0.001** | 93.37 (3.36) | **≤ 0.001a** |
|  | **2’FL** | 1.44 (0.10) | 13.59 (4.08) | **0.004** | 34.41 (4.85) | **≤ 0.001** | 44.42 (3.92) | **≤ 0.001b** |
|  | **OF/2’FL** | 1.43 (0.10) | 16.89 (3.68) | **≤ 0.001** | 55.94 (4.67) | **≤ 0.001** | 83.83 (5.56) | **≤ 0.001a** |
|  | **Negative** | 1.43 (0.10) | 1.58 (0.10) | 1.00 | 2.01 (0.23) | 1.00 | 2.51 (0.43) | 1.00c |
| ***P* (b)** | | 1.00 | **≤ 0.001** | | **≤ 0.001** | | **≤ 0.001** | |

| Propionate | Time (h) | T0 | T10 | | T24 | | T48 | |
| --- | --- | --- | --- | --- | --- | --- | --- | --- |
|  |  | **Mean (SE)** | **Mean (SE)** | ***P* (a)** | **Mean (SE)** | ***P* (a)** | **Mean (SE)** | ***P* (a)** |
| Substrate | **OF** | 0.89 (0.11) | 4.20 (0.46) | **≤ 0.001** | 22.72 (1.59) | **≤ 0.001** | 34.72 (2.06) | **≤ 0.001a** |
|  | **2’FL** | 0.90 (0.11) | 1.35 (0.47) | **≤ 0.001** | 5.77 (0.77) | **≤ 0.001** | 13.55 (1.91) | **≤ 0.001b** |
|  | **OF/2’FL** | 0.90 (0.12) | 5.08 (0.52) | **≤ 0.001** | 14.06 (1.40) | **≤ 0.001** | 32.11 (1.39) | **≤ 0.001a** |
|  | **Negative** | 0.90 (0.12) | 1.03 (0.13) | 1.00 | 1.14 (0.15) | 1.00 | 1.25 (0.15) | 1.00c |
| *P* (b) | | 1.00 | **≤ 0.001** | | **≤ 0.001** | | **≤ 0.001** | |

GC-FID analysis of acetate and propionate concentrations in the supernatant of effluents collected from vessel 1-4 at 0, 10, 24 and 48 h representing the mean (*n* = 5) and standard error (SE) of the data. Concentration reported in (mmol l^-1^). **(a)** Significant differences compared with respective 0 h sampling are identified with specified *P* values (grey columns). **(b)** Significant differences between substrates at 0, 10. 24 and 48 h are indicated by specified *P* values (orange row). Significant differences between substrates at 48 h are indicated by differing letters (grey column). **Abbreviations:** OF = oligofructose; 2’FL = 2’fucosyllactose

| **Butyrate** | **Time (h)** | **T0** | **T10** | | **T24** | | **T48** | |
| --- | --- | --- | --- | --- | --- | --- | --- | --- |
|  |  | **Mean (SE)** | **Mean (SE)** | ***P* (a)** | **Mean (SE)** | ***P* (a)** | **Mean (SE)** | ***P* (a)** |
| **Substrate** | **OF** | 0.63 (0.20) | 4.78 (0.93) | **≤ 0.001** | 16.95 (1.08) | **≤ 0.001** | 22.85 (1.62) | **≤ 0.001a** |
|  | **2’FL** | 0.62 (0.20) | 1.47 (0.87) | 1.00 | 4.65 (0.71) | **≤ 0.001** | 7.2 (1.28) | **≤ 0.001b** |
|  | **OF/2’FL** | 0.63 (0.20) | 4.43 (0.85) | **≤ 0.001** | 12.94 (1.12) | **≤ 0.001** | 19.28 (1.74) | **≤ 0.001a** |
|  | **Negative** | 0.63 (0.20) | 0.66 (0.22) | 1.00 | 0.75 (0.21) | 1.00 | 0.90 (0.22) | 1.00c |
| ***P* (b)** | | 1.00 | **0.003** | | **≤ 0.001** | | **≤ 0.001** | |

| Lactate | Time (h) | T0 | T10 | | T24 | | T48 | |
| --- | --- | --- | --- | --- | --- | --- | --- | --- |
|  |  | **Mean (SE)** | **Mean (SE)** | ***P* (a)** | **Mean (SE)** | ***P* (a)** | **Mean (SE)** | ***P* (a)** |
| Substrate | **OF** | 0 | 5.96 (0.24) | **≤ 0.001** | 4.16 (0.23) | **≤ 0.001** | 1.29 (0.12) | **≤ 0.001a** |
|  | **2’FL** | 0 | 4.01 (0.40) | **≤ 0.001** | 2.72 (0.75) | **≤ 0.001** | 0.54 (0.15) | **≤ 0.001a** |
|  | **OF/2’FL** | 0 | 4.77 (0.16) | **≤ 0.001** | 3.06 (0.22) | **≤ 0.001** | 0.94 (0.14) | **≤ 0.001a** |
|  | **Negative** | 0 | 0 | 1.00 | 0 | 1.00 | 0 | 1.00a |
| *P* (b) | | 1.00 | **≤ 0.001** | | **≤ 0.001** | | **≤ 0.001** | |

GC-FID analysis of butyrate and lactate concentrations in the supernatant of effluents collected from vessel 1-4 at 0, 10, 24 and 48 h representing the mean (*n* = 5) and standard error (SE) of the data. Concentration reported in (mmol l^-1^). **(a)** Significant differences compared with respective 0 h sampling are identified with specified *P* values (grey columns). **(b)** Significant differences between substrates at 0, 10. 24 and 48 h are indicated by specified *P* values (orange row). Significant differences between substrates at 48 h are indicated by differing letters (grey column). **Abbreviations:** OF = oligofructose; 2’FL = 2’fucosyllactose

| Succinate | Time (h) | T0 | T10 | | T24 | | T48 | |
| --- | --- | --- | --- | --- | --- | --- | --- | --- |
|  |  | **Mean (SE)** | **Mean (SE)** | ***P* (a)** | **Mean (SE)** | ***P* (a)** | **Mean (SE)** | ***P* (a)** |
| Substrate | **OF** | 0 | 0.13 (0.04) | **≤ 0.001** | 0.31(0.06) | **≤ 0.001** | 0.73 (0.08) | **≤ 0.001a** |
|  | **2’FL** | 0 | 0.07 (0.01) | **0.06** | 0.13 (0.04) | **0.06** | 0.27 (0.07) | **0.002b** |
|  | **OF/2’FL** | 0 | 0.10 (0.02) | **≤ 0.001** | 0.24 (0.05) | **≤ 0.001** | 0.6 (0.06) | **≤ 0.001a** |
|  | **Negative** | 0 | 0 | 1.00 | 0 | 1.00 | 0 | 1.00c |
|  | ***P* (b)** | 1.00 | **0.005** | | **≤ 0.001** | | **≤ 0.001** | |

| Total organic acids | Time (h) | T0 | T10 | | T24 | | T48 | |
| --- | --- | --- | --- | --- | --- | --- | --- | --- |
|  |  | **Mean (SE)** | **Mean (SE)** | ***P* (a)** | **Mean (SE)** | ***P* (a)** | **Mean (SE)** | ***P* (a)** |
| Substrate | **OF** | 2.94 (0.17) | 37.83 (5.19) | **≤ 0.001** | 97.95 (5.60) | **≤ 0.001** | 152.96 (5.55) | **≤ 0.001a** |
|  | **2’FL** | 2.95 (0.16) | 20.68 (4.46) | **0.01** | 47.88 (4.74) | **≤ 0.001** | 64.19 (4.10) | **≤ 0.001b** |
|  | **OF/2’FL** | 2.96 (0.16) | 29.02 (6.82) | **≤ 0.001** | 80.84 (4.70) | **≤ 0.001** | 136.76 (6.48) | **≤ 0.001a** |
|  | **Negative** | 2.96 (0.17) | 3.26 (0.28) | 1.00 | 3.90 (0.40) | 1.00 | 4.65 (0.58) | 1.00c |
| *P* (b) | | 1.00 | **0.005** | | **≤ 0.001** | | **≤ 0.001** | |

GC-FID analysis of succinate and total organic acid concentrations in the supernatant of effluents collected from vessel 1-4 at 0, 10, 24 and 48 h representing the mean (*n* = 5) and standard error (SE) of the data. Concentration reported in (mmol l^-1^). **(a)** Significant differences compared with respective 0 h sampling are identified with specified *P* values (grey columns). **(b)** Significant differences between substrates at 0, 10. 24 and 48 h are indicated by specified *P* values (orange row). Significant differences between substrates at 48 h are indicated by differing letters (grow column). **Abbreviations:** OF = oligofructose; 2’FL = 2’fucosyllactose

## **Supplemental Data Table 4 Individual organic acid data for all 5 donors used during *in vitro* fermentation**

| Acetate | | | | | | | | | | | | | | | | | | | | |
| --- | --- | --- | --- | --- | --- | --- | --- | --- | --- | --- | --- | --- | --- | --- | --- | --- | --- | --- | --- | --- |
| Time (h) | **OF** | | | | | **2’FL** | | | | | **OF/2’FL** | | | | | **Negative** | | | | |
| T0 | 1.51 | 1.41 | 1.51 | 1.66 | 1.05 | 1.52 | 1.42 | 1.52 | 1.66 | 1.05 | 1.52 | 1.42 | 1.51 | 1.67 | 1.06 | 1.52 | 1.41 | 1.51 | 1.67 | 1.06 |
| T10 | 22.80 | 18.90 | 24.70 | 27.03 | 22.35 | 3.39 | 13.41 | 21.70 | 5.77 | 23.68 | 11.96 | 18.75 | 5.28 | 23.73 | 24.72 | 1.86 | 1.43 | 1.57 | 1.72 | 1.31 |
| T24 | 76.39 | 77.23 | 58.59 | 65.98 | 56.91 | 38.24 | 37.59 | 48.89 | 22.19 | 25.14 | 45.46 | 73.08 | 54.82 | 55.99 | 50.34 | 2.79 | 2.23 | 1.70 | 1.86 | 1.47 |
| T48 | 102.76 | 94.43 | 91.50 | 82.15 | 96.03 | 53.24 | 44.02 | 50.25 | 30.45 | 44.14 | 102.76 | 84.85 | 83.31 | 68.16 | 80.07 | 2.98 | 1.99 | 3.98 | 1.87 | 1.72 |

Individual donor organic acid data – acetate concentrations across 0, 10, 24, and 48 h fermentation. Concentration in (mmol l^-1^). **Abbreviations:** OF = oligofructose; 2’FL = 2’fucosyllactose

| Propionate | | | | | | | | | | | | | | | | | | | | |
| --- | --- | --- | --- | --- | --- | --- | --- | --- | --- | --- | --- | --- | --- | --- | --- | --- | --- | --- | --- | --- |
| Time (h) | **OF** | | | | | **2’FL** | | | | | **OF/2’FL** | | | | | **Negative** | | | | |
| T0 | 0.78 | 1.19 | 0.82 | 0.56 | 1.09 | 0.78 | 1.20 | 0.83 | 0.58 | 1.09 | 0.79 | 1.23 | 0.83 | 0.57 | 1.10 | 0.78 | 1.23 | 0.83 | 0.57 | 1.09 |
| T10 | 5.17 | 3.04 | 3.48 | 3.98 | 5.35 | 3.02 | 0.35 | 1.03 | 0.67 | 1.69 | 5.15 | 5.65 | 4.18 | 3.75 | 6.64 | 1.31 | 1.24 | 0.84 | 0.63 | 1.11 |
| T24 | 20.42 | 21.54 | 22.51 | 20.26 | 28.86 | 8.04 | 5.18 | 5.50 | 3.44 | 6.68 | 15.71 | 10.66 | 11.66 | 13.87 | 18.42 | 1.68 | 1.11 | 0.97 | 0.78 | 1.15 |
| T48 | 31.04 | 33.48 | 37.11 | 30.46 | 41.50 | 10.64 | 15.21 | 15.69 | 7.75 | 18.43 | 30.98 | 30.40 | 33.76 | 28.76 | 36.65 | 1.79 | 1.23 | 1.25 | 0.87 | 1.11 |

Individual donor organic acid data – propionate concentrations across 0, 10, 24, and 48 h fermentation. Concentration in (mmol l^-1^). **Abbreviations:** OF = oligofructose; 2’FL = 2’fucosyllactose

| Butyrate | | | | | | | | | | | | | | | | | | | | |
| --- | --- | --- | --- | --- | --- | --- | --- | --- | --- | --- | --- | --- | --- | --- | --- | --- | --- | --- | --- | --- |
| Time (h) | **OF** | | | | | **2’FL** | | | | | **OF/2’FL** | | | | | **Negative** | | | | |
| T0 | 0.84 | 0.27 | 0.50 | 0.23 | 1.30 | 0.80 | 0.27 | 0.50 | 0.22 | 1.31 | 0.82 | 0.26 | 0.51 | 0.23 | 1.31 | 0.83 | 0.27 | 0.50 | 0.23 | 1.31 |
| T10 | 3.11 | 3.68 | 4.02 | 4.71 | 8.37 | 0.70 | 0.56 | 0.73 | 0.42 | 4.94 | 3.05 | 2.80 | 4.03 | 4.68 | 7.57 | 0.81 | 0.27 | 0.52 | 0.26 | 1.43 |
| T24 | 16.94 | 15.08 | 18.20 | 14.26 | 20.25 | 3.69 | 3.75 | 3.50 | 5.05 | 7.28 | 10.85 | 12.10 | 12.54 | 11.93 | 17.30 | 0.84 | 0.39 | 0.80 | 0.26 | 1.48 |
| T48 | 22.11 | 20.25 | 21.92 | 20.79 | 29.19 | 3.78 | 5.33 | 6.61 | 10.18 | 10.12 | 18.65 | 16.13 | 18.73 | 16.95 | 25.94 | 0.87 | 0.39 | 1.09 | 0.53 | 1.61 |

Individual donor organic acid data – butyrate concentrations across 0, 10, 24, and 48 h fermentation. Concentration in (mmol l^-1^). **Abbreviations:** OF = oligofructose; 2’FL = 2’fucosyllactose

| Lactate | | | | | | | | | | | | | | | | | | | | |
| --- | --- | --- | --- | --- | --- | --- | --- | --- | --- | --- | --- | --- | --- | --- | --- | --- | --- | --- | --- | --- |
| Time (h) | **OF** | | | | | **2’FL** | | | | | **OF/2’FL** | | | | | **Negative** | | | | |
| T0 | 0.00 | 0.00 | 0.00 | 0.00 | 0.00 | 0.00 | 0.00 | 0.00 | 0.00 | 0.00 | 0.00 | 0.00 | 0.00 | 0.00 | 0.00 | 0.00 | 0.00 | 0.00 | 0.00 | 0.00 |
| T10 | 6.29 | 6.37 | 5.11 | 6.32 | 5.71 | 3.72 | 3.21 | 4.72 | 5.18 | 3.23 | 4.78 | 4.21 | 5.19 | 4.82 | 4.84 | 0.00 | 0.00 | 0.00 | 0.00 | 0.00 |
| T24 | 4.16 | 3.42 | 4.79 | 4.45 | 3.97 | 1.41 | 1.32 | 5.22 | 3.62 | 2.05 | 3.03 | 2.40 | 3.80 | 3.05 | 3.02 | 0.00 | 0.00 | 0.00 | 0.00 | 0.00 |
| T48 | 1.41 | 0.92 | 1.19 | 1.32 | 1.62 | 0.20 | 0.30 | 0.56 | 0.63 | 1.03 | 0.90 | 0.57 | 0.87 | 0.95 | 1.42 | 0.00 | 0.00 | 0.00 | 0.00 | 0.00 |

Individual donor organic acid data – lactate concentrations across 0, 10, 24, and 48 h fermentation. Concentration in (mmol l^-1^), **Abbreviations:** OF = oligofructose; 2’FL = 2’fucosyllactosse

| Succinate | | | | | | | | | | | | | | | | | | | | |
| --- | --- | --- | --- | --- | --- | --- | --- | --- | --- | --- | --- | --- | --- | --- | --- | --- | --- | --- | --- | --- |
| Time (h) | **OF** | | | | | **2’FL** | | | | | **OF/2’FL** | | | | | **Negative** | | | | |
| T0 | 0.00 | 0.00 | 0.00 | 0.00 | 0.00 | 0.00 | 0.00 | 0.00 | 0.00 | 0.00 | 0.00 | 0.00 | 0.00 | 0.00 | 0.00 | 0.00 | 0.00 | 0.00 | 0.00 | 0.00 |
| T10 | 0.28 | 0.09 | 0.10 | 0.10 | 0.10 | 0.10 | 0.05 | 0.02 | 0.07 | 0.10 | 0.20 | 0.06 | 0.08 | 0.09 | 0.09 | 0.00 | 0.00 | 0.00 | 0.00 | 0.00 |
| T24 | 0.45 | 0.43 | 0.16 | 0.17 | 0.33 | 0.27 | 0.10 | 0.08 | 0.09 | 0.09 | 0.35 | 0.30 | 0.10 | 0.15 | 0.28 | 0.00 | 0.00 | 0.00 | 0.00 | 0.00 |
| T48 | 0.80 | 0.74 | 0.53 | 0.60 | 0.96 | 0.39 | 0.21 | 0.20 | 0.11 | 0.47 | 0.64 | 0.57 | 0.47 | 0.49 | 0.83 | 0.00 | 0.00 | 0.00 | 0.00 | 0.00 |

Individual donor organic acid data – succinate concentrations across 0, 10, 24, and 48 h fermentation. Concentration in (mmol l^-1^). **Abbreviations:** OF = oligofructose; 2’FL = 2’fucosyllactose

| Total organic acids | | | | | | | | | | | | | | | | | | | | |
| --- | --- | --- | --- | --- | --- | --- | --- | --- | --- | --- | --- | --- | --- | --- | --- | --- | --- | --- | --- | --- |
|  | **OF** | | | | | **2’FL** | | | | | **OF/2’FL** | | | | | **Negative** | | | | |
| T0 | 3.12 | 2.87 | 2.84 | 2.44 | 3.44 | 3.11 | 2.89 | 2.84 | 2.47 | 3.45 | 3.13 | 2.91 | 2.84 | 2.46 | 3.46 | 3.13 | 2.90 | 2.84 | 2.46 | 3.46 |
| T10 | 35.65 | 28.07 | 32.41 | 35.14 | 57.89 | 10.92 | 18.58 | 28.23 | 12.09 | 33.60 | 23.14 | 25.28 | 12.76 | 30.07 | 53.85 | 3.99 | 2.93 | 2.93 | 2.61 | 3.86 |
| T24 | 104.37 | 97.70 | 84.25 | 88.13 | 115.32 | 51.65 | 47.94 | 63.19 | 35.39 | 41.23 | 71.40 | 91.54 | 77.91 | 73.99 | 89.36 | 5.31 | 3.73 | 3.46 | 2.91 | 4.11 |
| T48 | 158.12 | 149.82 | 152.25 | 135.31 | 169.31 | 69.25 | 65.07 | 73.31 | 49.12 | 64.20 | 153.94 | 132.52 | 137.13 | 115.31 | 144.91 | 5.63 | 3.61 | 6.32 | 3.27 | 4.44 |

Individual donor organic acid data – total SCFA concentrations across 0, 10, 24, and 48 h fermentation. Concentration in (mmol l^-1^). **Abbreviations:** OF = oligofructose; 2’FL = 2’fucosyllactose

## **Supplemental Data Table 5 Mean bacterial populations using pH-controlled *in vitro* batch culture fermentation at 0, 10, 24, and 48 h**

| Total Bacteria (Eub I-II-III) | Time (h) | T0 | T10 | | T24 | | T48 | |
| --- | --- | --- | --- | --- | --- | --- | --- | --- |
|  |  | **Mean (SE)** | **Mean (SE)** | ***P* (a)** | **Mean (SE)** | ***P* (a)** | **Mean (SE)** | ***P* (a)** |
| Substrate | **OF** | 8.24 (0.10) | 8.84 (0.11) | **0.003** | 8.95 (0.06) | **0.003** | 8.50 (0.03) | 0.62 |
|  | **2’FL** | 8.25 (0.1) | 8.67 (0.6) | **0.046** | 8.76 (0.04) | **0.034** | 8.31 (0.07) | 1.00 |
|  | **OF/2’FL** | 8.25 (0.1) | 8.73 (0.05) | **0.017** | 8.80 (0.06) | **0.021** | 8.42 (0.10) | 1.00 |
|  | **Negative** | 8.24 (0.1) | 7.97 (0.12) | 0.56 | 7.97 (0.02) | 0.67 | 7.81 (0.13) | 0.08 |
| *P* (b) | | 1.00 | **≤ 0.001** | | **≤ 0.001** | | **≤ 0.001** | |

| *Bifidobacterium* spp. (Bif164) | Time (h) | T0 | T10 | | T24 | | T48 | |
| --- | --- | --- | --- | --- | --- | --- | --- | --- |
|  |  | **Mean (SE)** | **Mean (SE)** | ***P* (a)** | **Mean (SE)** | ***P* (a)** | **Mean (SE)** | ***P* (a)** |
| Substrate | **OF** | 7.37 (0.10) | 8.67 (0.15) | **≤ 0.001** | 8.85 (0.07) | **≤ 0.001** | 8.25 (0.07) | **≤ 0.001** |
|  | **2’FL** | 7.37 (0.10) | 8.50 (0.10) | **≤ 0.001** | 8.60 (0.07) | **≤ 0.001** | 8.06 (0.04) | **≤ 0.001** |
|  | **OF/2’FL** | 7.36 (0.10) | 8.51 (0.08) | **0.002** | 8.63 (0.06) | **0.002** | 8.21 (0.08) | **0.002** |
|  | **Negative** | 7.37 (0.10) | 7.37 (0.16) | 1.00 | 7.42 (0.15) | 1.00 | 7.33 (0.18) | **1.00b** |
| *P* (b) | | 1.00 | **≤ 0.001** | | **≤ 0.001** | | **≤ 0.001** | |

Bacterial groups measured by FISH-FLOW (Log10 cells/mL) using probe: Total bacteria (Eub I-II-II) and *Bifidobacterium* (Bif164) collected at 0, 10, 24 and 48 h representing the mean (*n* = 5) and standard error (SE) of the data. **(a)** Significant differences compared with respective 0 h sampling are identified with specified *P* values (grey columns). **(b)** Significant differences between substrates at 0, 10. 24 and 48 h are indicated by specified *P* values (orange rows). **Abbreviations:** OF = oligofructose; 2’FL = 2’fucosyllactose

| Most *Bacteroidaceae* and *Prevotellaceae*, some *Porphyromonadaceae* (Bac303) | Time (h) | T0 | T10 | | T24 | | T48 | |
| --- | --- | --- | --- | --- | --- | --- | --- | --- |
|  |  | **Mean (SE)** | **Mean (SE)** | ***P* (a)** | **Mean (SE)** | ***P* (a)** | **Mean (SE)** | ***P* (a)** |
| Substrate | **OF** | 6.19 (0.18) | 7.35 (0.15) | **≤ 0.001** | 7.83 (0.07) | **≤ 0.001** | 7.56 (0.09) | **≤ 0.001a** |
|  | **2’FL** | 6.20 (0.16) | 7.05 (0.08) | **0.01** | 7.15 (0.15) | **0.01** | 6.87 (0.19) | 0.09b |
|  | **OF/2’FL** | 6.17 (0.17) | 7.38 (0.07) | **≤ 0.001** | 7.49 (0.20) | **≤ 0.001** | 7.24 (0.13) | **0.003ab** |
|  | **Negative** | 6.18 (0.16) | 6.19 (0.18) | 1.00 | 6.12 (0.14) | 1.00 | 5.79 (0.18) | 0.81c |
| *P* (b) | | 1.00 | **≤ 0.001** | | **≤ 0.001** | | **≤ 0.001** | |

| *Lactobacillus /Enterococcus* (Lab158) | Time (h) | T0 | T10 | | T24 | | T48 | |
| --- | --- | --- | --- | --- | --- | --- | --- | --- |
|  |  | **Mean (SE)** | **Mean (SE)** | ***P* (a)** | **Mean (SE)** | ***P* (a)** | **Mean (SE)** | ***P* (a)** |
| Substrate | **OF** | 5.97 (0.09) | 6.78 (0.14) | **≤ 0.001** | 6.67 (0.14) | **0.006** | 6.25 (0.22) | 1.00a |
|  | **2’FL** | 5.96 (0.07) | 6.72 (0.18) | **0.002** | 6.70 (0.23) | **0.004** | 6.34 (0.22) | 0.43a |
|  | **OF/2’FL** | 5.91 (0.09) | 6.33 (0.21) | **0.013** | 6.61 (0.21) | **0.006** | 6.39 (0.21) | 0.18a |
|  | **Negative** | 5.92 (0.07) | 5.96 (0.05) | 1.00 | 5.94 (0.08) | 1.00 | 5.65 (0.15) | 1.00a |
| *P* (b) | | 1.00 | **0.006** | | **0.02** | | 0.69 | |

Bacterial groups measured by FISH-FLOW (Log10 cells/mL) using probe: most *Bacteroidacae* and *Prevotellaceae* (Bac303) and Lactobacillus/*Enterococcus* (Lab158) collected at 0, 10, 24 representing the mean (*n* = 5) and standard error (SE) of the data. **(a)** Significant differences compared with respective 0 h sampling are identified with specified *P* values (grey columns). **(b)** Significant differences between substrates at 0, 10. 24 and 48 h are indicated by specified *P* values (orange column). Significant differences between substrates at 48h are indicated by differing letters (grey column). **Abbreviations:** OF = oligofructose; 2’FL = 2’fucosyllactose

## **Supplemental Data Table 6 Individual bacterial populations using pH-controlled *in vitro* batch culture fermentation at 0, 10, 24, and 48 h**

| Total Bacteria (Eub338 I-II-III) | | | | | | | | | | | | | | | | | | | | |
| --- | --- | --- | --- | --- | --- | --- | --- | --- | --- | --- | --- | --- | --- | --- | --- | --- | --- | --- | --- | --- |
| Time (h) | **OF** | | | | | **2’FL** | | | | | **OF/2’FL** | | | | | **Negative** | | | | |
| T0 | 8.57 | 8.34 | 8.12 | 8.09 | 8.06 | 8.58 | 8.34 | 8.16 | 8.1 | 8.06 | 8.59 | 8.33 | 8.16 | 8.09 | 8.06 | 8.58 | 8.33 | 8.15 | 8.1 | 8.06 |
| T10 | 8.75 | 8.58 | 8.72 | 9.15 | 9.02 | 8.68 | 8.8 | 8.51 | 8.57 | 8.79 | 8.66 | 8.6 | 8.78 | 8.77 | 8.86 | 8.08 | 8.23 | 7.68 | 7.77 | 8.22 |
| T24 | 8.82 | 8.91 | 8.86 | 9.16 | 9.01 | 8.78 | 8.67 | 8.75 | 8.7 | 8.92 | 8.8 | 8.62 | 8.84 | 8.77 | 8.96 | 7.93 | 8.13 | 7.63 | 7.51 | 8.66 |
| T48 | 8.4 | 8.45 | 8.52 | 8.57 | 8.57 | 8.23 | 8.11 | 8.43 | 8.31 | 8.48 | 8.19 | 8.23 | 8.62 | 8.4 | 8.66 | 7.81 | 8.11 | 7.55 | 7.51 | 8.08 |

Bacterial groups measured by FISH-FLOW (Log10 cells/mL) using probe: total bacteria (Eub338 I-II-III) at 0, 10 , 24 and 48 h. **Abbreviations:** OF = oligofructose; 2’FL = 2’fucosyllactose

| *Bifidobacterium* spp. (Bif164) | | | | | | | | | | | | | | | | | | | | |
| --- | --- | --- | --- | --- | --- | --- | --- | --- | --- | --- | --- | --- | --- | --- | --- | --- | --- | --- | --- | --- |
| Time (h) | **OF** | | | | | **2’FL** | | | | | **OF/2’FL** | | | | | **Neg** | | | | |
| T0 | 7.45 | 7.65 | 7.31 | 7.04 | 7.38 | 7.46 | 7.65 | 7.32 | 7.05 | 7.38 | 7.46 | 7.65 | 7.29 | 7.04 | 7.38 | 7.45 | 7.65 | 7.31 | 7.05 | 7.38 |
| T10 | 8.68 | 8.90 | 8.66 | 9.01 | 8.12 | 8.52 | 8.80 | 8.46 | 8.57 | 8.17 | 8.52 | 8.50 | 8.73 | 8.56 | 8.24 | 7.34 | 7.85 | 7.20 | 6.92 | 7.54 |
| T24 | 8.79 | 8.99 | 8.86 | 9.01 | 8.62 | 8.62 | 8.67 | 8.75 | 8.60 | 8.36 | 8.75 | 8.59 | 8.76 | 8.59 | 8.45 | 7.40 | 7.85 | 7.46 | 6.90 | 7.52 |
| T48 | 8.21 | 8.32 | 8.19 | 8.47 | 8.08 | 8.20 | 8.03 | 8.05 | 7.98 | 8.02 | 8.14 | 8.16 | 8.52 | 8.08 | 8.15 | 7.32 | 7.83 | 7.34 | 6.73 | 7.44 |

Bacterial groups measured by FISH-FLOW (Log10 cells/mL) using probe: *Bifidobacterium* spp. (Bif164) at 0, 10 , 24 and 48 h. **Abbreviations:** OF = oligofructose; 2’FL = 2’fucosyllactose

| Most *Bacteroidaceae* and *Prevotellaceae*, some *Porphyromonadaceae* (Bac303) | | | | | | | | | | | | | | | | | | | | | |
| --- | --- | --- | --- | --- | --- | --- | --- | --- | --- | --- | --- | --- | --- | --- | --- | --- | --- | --- | --- | --- | --- |
| Time (h) | | **OF** | | | | | **2’FL** | | | | | **OF/2’FL** | | | | | **Neg** | | | | |
| T0 | 6.76 | | 6.44 | 5.94 | 5.94 | 5.87 | 6.75 | 6.38 | 6.06 | 5.95 | 5.87 | 6.74 | 6.41 | 5.88 | 5.95 | 5.87 | 6.71 | 6.36 | 6.03 | 5.95 | 5.87 |
| T10 | 6.99 | | 7.09 | 7.35 | 7.79 | 7.55 | 7.01 | 7 | 6.81 | 7.08 | 7.33 | 7.13 | 7.37 | 7.5 | 7.47 | 7.42 | 6.66 | 6.29 | 5.56 | 6.3 | 6.12 |
| T24 | 7.75 | | 7.65 | 7.92 | 7.79 | 8.04 | 6.99 | 6.93 | 7.72 | 6.99 | 7.12 | 7.12 | 7.04 | 7.88 | 7.37 | 8.05 | 6.65 | 6.16 | 5.91 | 5.85 | 6.01 |
| T48 | 7.65 | | 7.55 | 7.72 | 7.2 | 7.68 | 6.46 | 6.44 | 7.45 | 6.92 | 7.1 | 7.03 | 7.01 | 7.7 | 7.13 | 7.35 | 6.09 | 6.15 | 5.56 | 5.18 | 5.98 |

Bacterial groups measured by FISH-FLOW (Log10 cells/mL) using probes: most *Bacteroidacae* and *Prevotellaceae* (Bac303) at 0, 10 , 24 and 48 h. **Abbreviations:** OF = oligofructose; 2’FL = 2’fucosyllactose

| *Lactobacillus/Enterococcus* (Lab158) | | | | | | | | | | | | | | | | | | | | |
| --- | --- | --- | --- | --- | --- | --- | --- | --- | --- | --- | --- | --- | --- | --- | --- | --- | --- | --- | --- | --- |
| Time (h) | **OF** | | | | | **2’FL** | | | | | **OF/2’FL** | | | | | **Neg** | | | | |
| T0 | 5.71 | 6.2 | 5.81 | 6.11 | 6.02 | 5.7 | 5.96 | 5.99 | 6.11 | 6.02 | 5.67 | 6.04 | 5.7 | 6.11 | 6.02 | 5.7 | 5.93 | 5.86 | 6.11 | 6.02 |
| T10 | 6.31 | 6.65 | 6.84 | 7.17 | 6.92 | 6.23 | 6.39 | 6.93 | 6.89 | 7.17 | 5.74 | 6.47 | 6.16 | 7 | 6.27 | 5.78 | 6.06 | 5.98 | 5.98 | 6 |
| T24 | 6.94 | 6.31 | 6.66 | 7.02 | 6.41 | 6.39 | 6.24 | 6.86 | 7.5 | 6.49 | 6.15 | 6.26 | 6.6 | 7.37 | 6.65 | 5.79 | 5.97 | 6.2 | 5.73 | 6.01 |
| T48 | 6.22 | 5.45 | 6.46 | 6.82 | 6.3 | 6.52 | 5.88 | 6.18 | 7.13 | 6.01 | 6.04 | 6.15 | 6.2 | 7.19 | 6.35 | 5.25 | 5.95 | 5.43 | 5.61 | 6.00 |

Individual bacterial groups measured by FISH-FLOW (Log10 cells/mL) using probe: *Lactobacillus/Enterococcus* spp. (Lab158) at 0, 10 , 24 and 48 h. **Abbreviations:** OF = oligofructose; 2’FL = 2’fucosyllactose
